# A Ubiquitous Chromatin Opening Element and DNA Demethylation Facilitate Doxycycline-Controlled Expression during Differentiation and in Transgenic Mice

**DOI:** 10.1021/acssynbio.2c00450

**Published:** 2023-02-09

**Authors:** Natascha Gödecke, Sabrina Herrmann, Viola Weichelt, Dagmar Wirth

**Affiliations:** † RG Model Systems for Infection and Immunity, Helmholtz Centre for Infection Research, 38124 Braunschweig, Germany; ‡ Institute of Experimental Hematology, Medical University Hannover (MHH), 30625 Hannover, Germany

**Keywords:** inducible gene expression, Tet-on system, expression silencing during differentiation, UCOE, transgenic mice

## Abstract

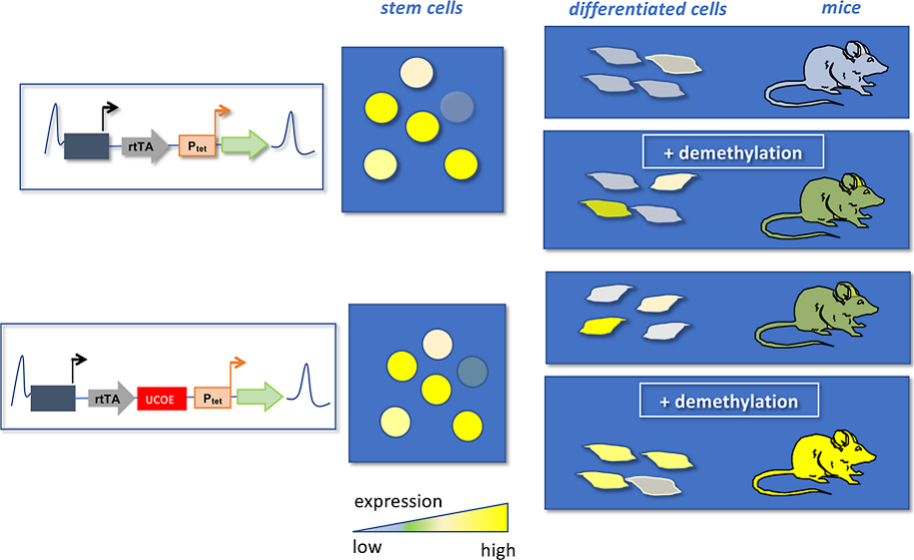

Synthetic expression cassettes provide the ability to control transgene expression in experimental animal models through external triggers, enabling the study of gene function and the modulation of endogenous regulatory networks in vivo. The performance of synthetic expression cassettes in transgenic animals critically depends on the regulatory properties of the respective chromosomal integration sites, which are affected by the remodeling of the chromatin structure during development. The epigenetic status may affect the transcriptional activity of the synthetic cassettes and even lead to transcriptional silencing, depending on the chromosomal sites and the tissue. In this study, we investigated the influence of the ubiquitous chromosome opening element (UCOE) HNRPA2B1-CBX3 and its subfragments A2UCOE and CBX3 on doxycycline-controlled expression modules within the chromosomal Rosa26 locus. While HNRPA2B1-CBX3 and A2UCOE reduced the expression of the synthetic cassettes in mouse embryonic stem cells, CBX3 stabilized the expression and facilitated doxycycline-controlled expression after in vitro differentiation. In transgenic mice, the CBX3 element protected the cassettes from overt silencing although the expression was moderate and only partially controlled by doxycycline. We demonstrate that CBX3-flanked synthetic cassettes can be activated by decitabine-mediated blockade of DNA methylation or by specific recruitment of the catalytic demethylation domain of the ten–eleven translocation protein TET1 to the synthetic promoter. This suggests that CBX3 renders the synthetic cassettes permissive for subsequent epigenetic activation, thereby supporting doxycycline-controlled expression. Together, this study reveals a strategy for overcoming epigenetic constraints of synthetic expression cassettes, facilitating externally controlled transgene expression in mice.

## Introduction

Synthetic expression cassettes have proven to be a powerful tool for external control of transgene expression^1^ and for dynamic modulation of endogenous gene expression in mammalian cells.^2^ The tetracycline system represents one of the first synthetic regulation systems^3^ and is widely used in various species. It is based on a synthetic P_tet_ promoter and a co-expressed cognate transcriptional activator (tTA or rtTA), which comprises a specific P_tet_ promoter-binding domain as well as a transcriptional activation domain. Transcription is initiated when the transcription activator rtTA or tTA binds to the cognate P_tet_ promoter, while binding crucially depends on the presence and absence of doxycycline, respectively.

Upon stable integration of expression cassettes into the host DNA, the performance of synthetic promoters and, in particular, the regulation potential, is affected by the chromosomal environment. Based on the mammalian genome architecture with its compartmentalization into various chromatin domains,^4,5^ cellular regulatory DNA elements such as enhancers, insulators, or silencers and the epigenetic status of a particular chromatin domain can influence the synthetic expression cassettes. Since standard gene transfer methods rely on random integration of transfected DNA, cell clones with tight regulation of the transgenes have to be identified by appropriate selection and sorting strategies.^6−8^ Since screening is limited for the establishment of transgenic mice, the specific targeting of cassettes into predefined chromosomal loci is a favorable strategy for prediction of expression properties in tissues.

For the generation of genetically modified mice, the well-characterized Rosa26 locus on chromosome 6 has been exploited for the integration of various transgenes and also recombinant expression cassettes controlled by heterologous promoters. While the Rosa26 promoter is ubiquitously active^9^ and targeted integration of various constitutive promoters into this chromosomal site conferred expression in many tissues,^10,11^ integration of the synthetic Tet cassettes resulted in variable expression in mice, with heterogeneity in between organs but also in between individual cells, resulting in an overall poor predictability of transgene expression.^12−14^ Previous studies showed that the P_tet_ promoter can be silenced by DNA methylation even when integrated into the ubiquitously accessible Rosa26 locus.^13^ Together, the variable performance of synthetic cassettes represents a challenge for implementing synthetic strategies in animal systems and hampers the generation of mice for controlled transgene expression.^14^

In the last decades, various chromosomal elements were identified that affect the chromatin status and modulate the expression of heterologous transcription units in cis. This includes scaffold-/matrix-associated regions,^15,16^ stabilizing anti-repressor elements,^17^ locus control regions, and insulator elements.^18−21^ More recently, so-called ubiquitous chromatin opening elements (UCOEs) were identified from few chromosomal sites, including the locus encoding the mouse ribosomal protein S3 (Rps3)^22^ as well as the human loci TBP-PSNB1 and HNRPA2B1/CBX3.^23^ UCOEs were shown to confer a favorable chromatin environment by preventing heterochromatin-mediated silencing^23^ and promote histone acetylation, which is associated with transcriptional activity.^24,25^ Based on these properties, UCOEs were exploited to prevent silencing of therapeutic genes in stem cells^26−29^ and also for recombinant protein production.^22,30−33^ So far, the potential of UCOEs was investigated in vitro, while its ability to overcome the silencing of synthetic cassettes in transgenic mice has not been explored.

In this study, we investigated if UCOEs can stabilize P_tet_-controlled expression in the Rosa26 locus, which was previously shown to silence the synthetic cassettes.^13^ We demonstrate that a subfragment of the HNRPA2B1/CBX3 UCOE, CBX3, is neutral for P_tet_-controlled expression in mouse embryonic stem (ES) cells but can reduce silencing of P_tet_ cassettes during differentiation. Moreover, CBX3 provides susceptibility to the decitabine-mediated block of DNA methylation as well as to active demethylation, thereby supporting Doxycycline-induced expression of the synthetic cassettes in transgenic mice.

## Results

### CBX3 UCOE Subfragment but Not the Full-Length HNRPA2B1-CBX3 UCOE Supports the Expression of the P_tet_ Promoter in the Rosa26 Locus of Mouse ES Cells

**Figure 1:**
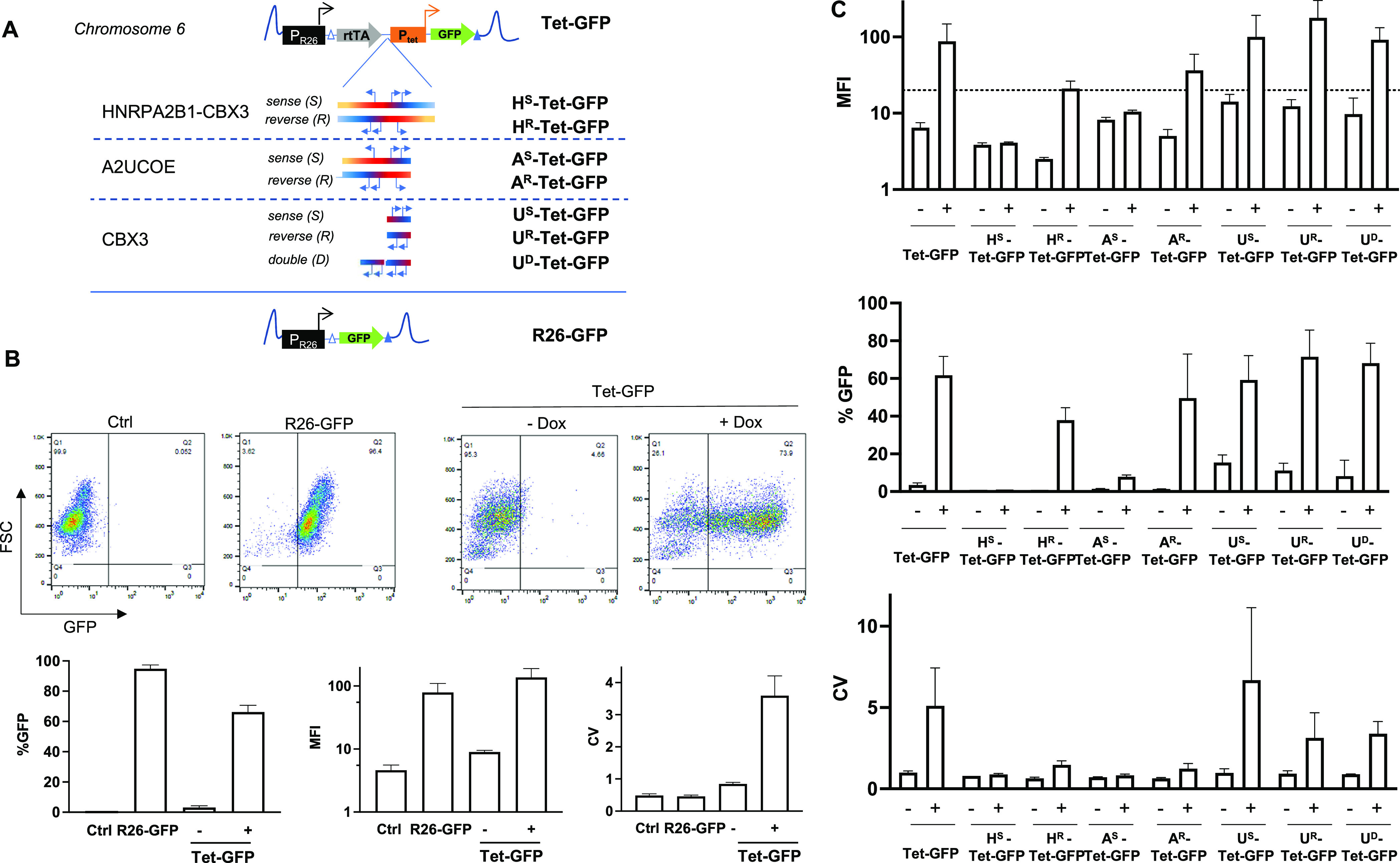
Influence of HNRPA2B1 UCOE and its subfragments on the synthetic P_tet_ promoter after targeted integration into the Rosa26 locus of ES cells. (A) Schematic drawing of the expression cassettes integrated into cells used in Figures 1 and 2. In Tet-GFP cells, the reverse transactivator (rtTA) (Tet-ON) is controlled by the endogenous Rosa26 promoter, while the P_tet_ promoter drives the expression of GFP. H^S^-Tet-GFP and H^R^-Tet-GFP cells harbor the 4kb HNRPA2B1-CBX3 UCOE, A^S^-Tet-GFP, and A^R^-Tet-GFP cells harbor the 1.5 kb A2UCOE, while U^S^-Tet-GFP and U^R^-Tet-GFP cells harbor the 0,7 kb CBX3 in the sense and reverse orientations, respectively. Blue arrows denote the position of three known endogenous promoters within the HNRPA2B1-CBX3 UCOE and its subfragments. In U^D^-Tet-GFP cells, two copies of the CBX3 UCOE are integrated in the reverse orientation. R26-GFP cells represent control cells in which GFP expression is driven by the endogenous Rosa26 promoter. All cassettes are flanked by heterospecific FRT sites (indicated by white and blue triangles) and were targeted into the murine Rosa26 locus on chromosome 6 by RMCE (see the Methods section for details). (B) GFP expression analysis in Tet-GFP and R26-GFP ES cells. The cells were cultivated in the absence or presence of 2 μg/mL doxycycline for 48 h and analyzed for GFP expression by flow cytometry. Representative flow cytometry plots of the indicated ES cells and non-modified control cells are depicted. The percentage of GFP-expressing cells (%), MFI, and robust CV are indicated below. Bars represent cumulative data from 2–3 experiments performed in duplicate or triplicate. (C) Expression analyses of the various ES cell populations. The cell populations were cultivated in the absence or presence of 2 μg/mL doxycycline for 48 h and analyzed for GFP expression by flow cytometry. MFI, percentage of GFP expression (%), and robust CV are displayed. Cumulative data from 1–3 experiments performed in duplicate or triplicate are represented. The dashed line reflects the background fluorescence (MFI) of control (non-modified) ES cells. Supporting Information Figure S1 provides underlying primary data in the presence and absence of doxycycline.

We based our study on clonal Tet-GFP mouse ES cells in which the endogenous Rosa26 promoter drives the constitutive expression of the synthetic, doxycycline-controlled transactivator rtTA (Tet-ON), while the neighboring P_tet_ promoter controls the expression of the green fluorescent protein (GFP) reporter (Figure 1A). This cassette design was previously shown to be prone to silencing during in vitro differentiation of ES cells and in transgenic animals derived thereof.^13^ A clonal population of Tet-GFP ES cells showed GFP expression in about 65% or 4% of cells when cultured in the presence or absence of doxycycline, respectively (Figure 1B). Notably, GFP expression was heterogeneous in between the isogenic cells, and the fluorescence intensity varied by about 2 orders of magnitude among expressing cells. In contrast, for control R26-GFP cells, in which the Rosa26 promoter drives the expression of GFP (Figure 1A), more than 95% of cells were positive for GFP, with high homogeneity in individual cells (Figure 1B). To quantify cell-to-cell expression heterogeneity, we assessed the robust coefficient of variation (CV). The robust CV is determined by the standard deviation divided by the median. It represents a statistic measure of the distribution around the median and reflects the variation among individual cells. While the control R26-GFP cells showed low variation with a CV of 0.5, doxycycline-induced expression in Tet-GFP reached a CV of about 3.5.

We evaluated if UCOEs could improve the homogeneity of P_tet_-controlled transgene expression. To this end, we focused on the 4kb UCOE derived from the HNRPA2B1-CBX3 locus as well as its 1.5 and 0.7 kb subfragments, designated A2UCOE and CBX3, respectively.^23,28,31,34^ Using Flp recombinase-mediated cassette exchange (RMCE),^35^ we generated clonal ES cell lines in which the various UCOE fragments were integrated into the Rosa26 locus in sense or in a reverse orientation upstream of the P_tet_ promoter (Figure 1A). Notably, integration of the HNRPA2B1-CBX3 UCOE (H^R/S^-Tet-GFP) and the A2UCOE (A^R/S^-Tet-GFP) reduced the GFP expression irrespective of the orientation, as evident from the median fluorescence intensity (MFI) and expression frequencies, suggesting a negative impact on the performance of the P_tet_ promoter in the Rosa26 locus (Figure 1C). In contrast, the integration of the CBX3 UCOE (U^R/S^-Tet-GFP) restored the expression of the UCOE free cassettes irrespective of the orientation, while integration of an additional CBX3 copy (U^D^-Tet-GFP) did not further improve the GFP expression. Notably, the integration of CBX3 stabilized the expression, while it did not improve the homogeneity of expression, as reflected by comparably high CVs (Figure 1C and Supporting Information Figure S1).

Together, this shows that the HNRPA2B1-CBX3 UCOE and its subfragment A2UCOE have a negative influence on P_tet_-controlled expression in the Rosa26 locus in ES cells. In contrast, the integration of the short CBX subfragment maintains the Doxycycline-controlled expression in ES cells, albeit without improving the expression level and homogeneity.

### CBX3 UCOE Protects the P_tet_ Promoter from Silencing during In Vitro Differentiation

**Figure 2:**
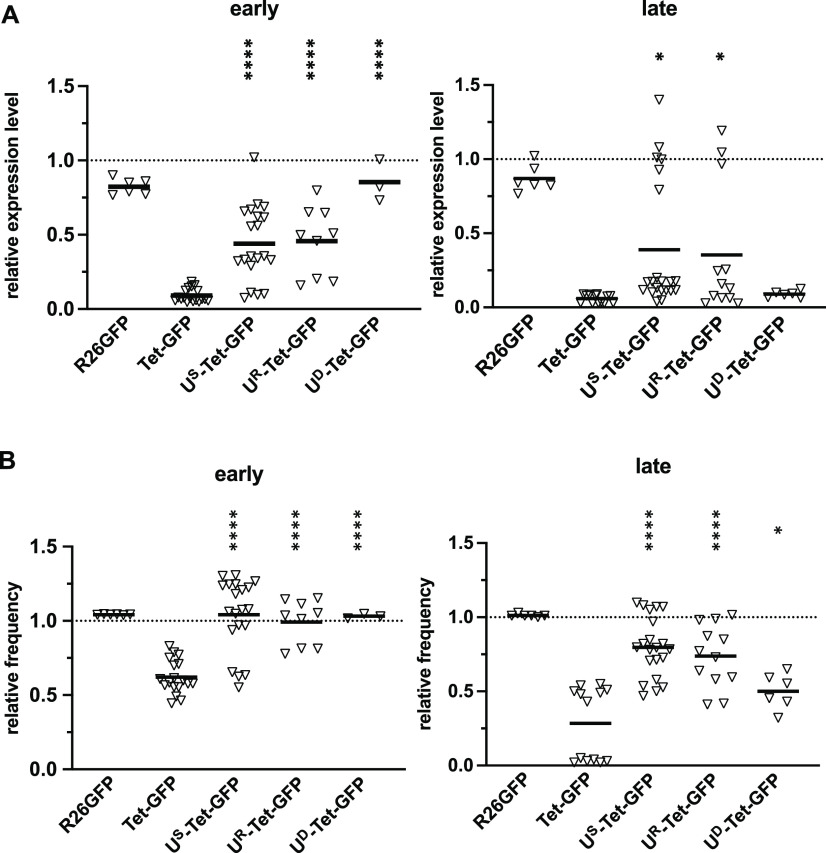
The CBX3 UCOE protects the P_tet_ promoter from silencing during differentiation in vitro. ES cells were differentiated by cultivation in the absence of LIF for 4 (early) and 8 (late) days, either in the presence or in the absence of 2 μg/mL doxycycline and subsequently analyzed by flow cytometry. Relative expression levels (MFI) (A) and relative frequency of GFP expressing cells (B) for + Dox cultures are depicted, in which the expression of the respective ES cell population was set to 1 (indicated by dashed lines). The individual data points represent samples from 1–3 experiments performed in triplicate or duplicate. Student’s *t*-test was used for comparisons between Tet-GFP and U^S^-Tet-GFP, U^R^-Tet-GFP, or U^D^-Tet-GFP, respectively, and is indicated above the respective data set. *p < 0.05; ****p < 0.0001. Supporting Information Figure S2 provides the underlying primary data in the presence and absence of doxycycline.

To investigate the expression properties after differentiation, we cultivated U^S^-Tet-GFP, U^R^-Tet-GFP, and U^D^-Tet-GFP cells in the absence of feeders and the stem cell leukemia inhibitory factor (LIF) and assessed GFP expression by flow cytometry on day 4 (early) and day 8 (late) of differentiation. As control, we employed R26-GFP cells. R26-GFP cells largely maintained high expression during differentiation with regard to the overall MFI (Figure 2A) and expression frequency (Figure 2B). In contrast, differentiated Tet-GFP cells showed a 20-fold reduction in GFP expression, with a relative frequency of about 60% GFP-positive cells at day 4. On day 8, silencing was even more pronounced with a lack of expression in about half of the samples and very low expression in the remaining samples. This heterogeneity in independent samples suggests a certain level of stochasticity in differentiation-associated silencing. In contrast, in differentiated U^S^-Tet-GFP and U^R^-Tet-GFP cells, GFP expression levels were reduced to a lower degree on day 4 and dropped down to 20–30% on day 8 in the majority of samples (Figure 2A). The integration of an additional copy of CBX3 in U^D^-Tet-GFP cells further delayed but could not prevent silencing of the cassettes at late time points. Notably, also in differentiated cells, the CBX3 element did not compromise doxycycline control and expression was efficiently switched off in the absence of doxycycline (Supporting Information Figure S2). Together, this demonstrates that CBX3 can reduce but does not completely overcome silencing during in vitro differentiation.

### CBX3 UCOE Facilitates P_tet_-Luc Expression In Vivo

**Figure 3:**
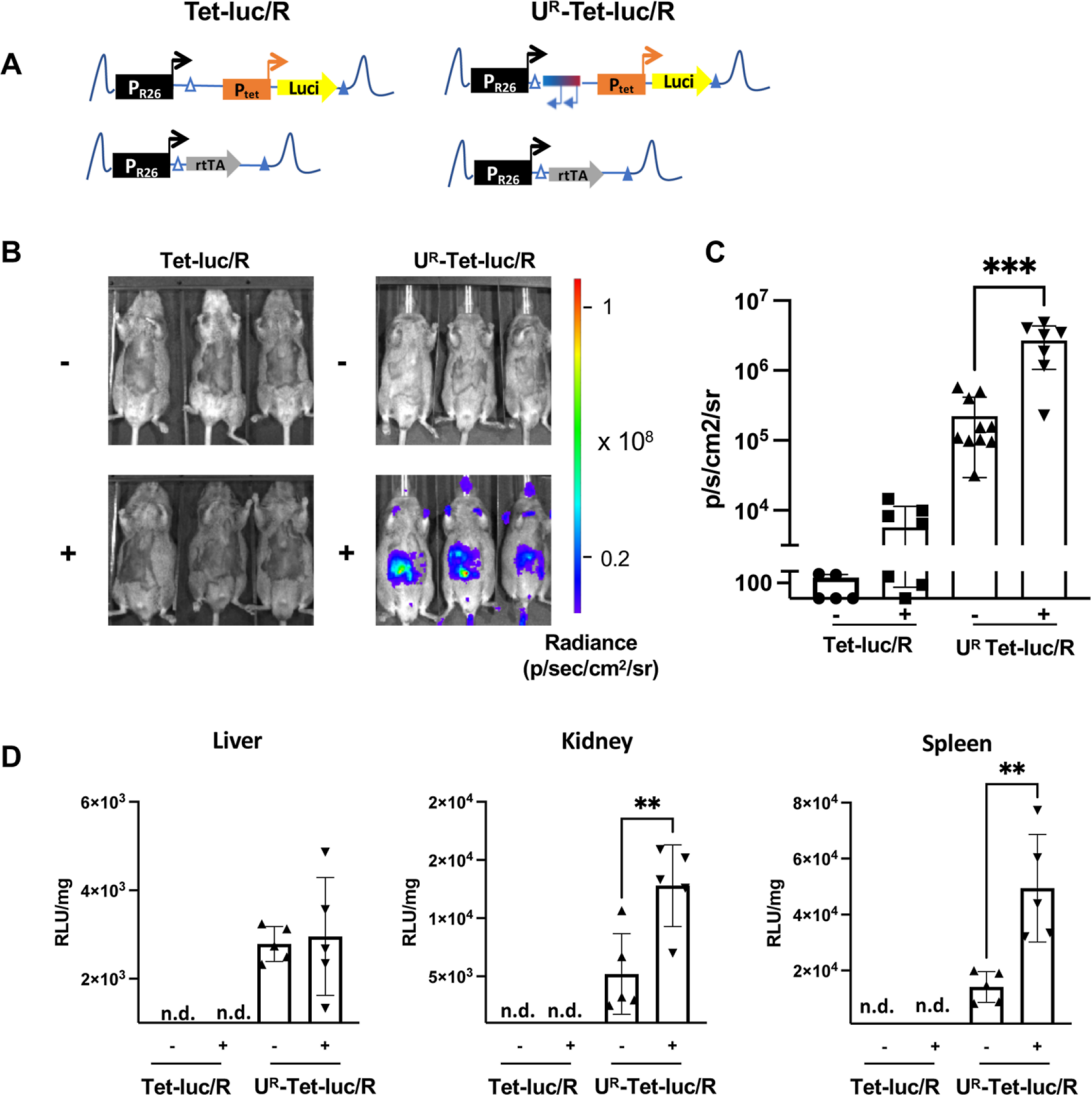
The CBX3 UCOE protects the Tet promoter from being silenced in vivo. (A) Schematic drawing of the synthetic cassettes targeted into the Rosa26 locus in transgenic mice. Tet-luc/R and U^R^-Tet-Luc/R mice encode a P_tet_ promoter-driven fusion protein of luciferase and GFP integrated in the Rosa26 locus as well as a Rosa26 promoter-driven rtTA on the homologous chromosome. In U^R^-Tet-Luc/R a reversely orientated CBX3 UCOE is integrated upstream of the P_tet_ promoter. White and blue triangles indicated heterologous FRT sites used for targeted integration of the cassettes into the murine Rosa26 locus on chromosome 6. (B) Representative in vivo non-invasive bioluminescent images of induced (+) or uninduced (−) Tet-luc/R or U^R^-Tet-luc/R mice. The mice received 2 mg/mL doxycycline administration in drinking water for 3 weeks or were left untreated. The color bar indicates photons/cm^2^/s/steradian. (C) The graph shows the quantification of the BLI images based on 5 Tet-luc/R and 10 U^R^-Tet-luc/R mice in the absence of doxycycline, and 7 Tet-luc/R and 7 U^R^-Tet-luc/R mice received doxycycline for 21 days. Student’s *t*-test was used for statistical analysis. ***p < 0.001. (D) Organ lysates were prepared from livers, kidneys, and spleens of Tet-luc/R and U^R^-Tet-luc/R mice (treated with doxycycline for 21 days or left untreated) and analyzed for the luciferase expression. The luciferase activity (RLU) was normalized to milligrams of total protein in the tissue sample. The data were derived from 5 Tet-luc/R and 5 U^R^-Tet-luc/R mice in the off state and from 7 Tet-luc/R and 5 U^R^-Tet-luc/R transgenic animals in the on state. n.d.: all samples were below the detection limit or <1000 RLU/mg. Student’s *t*-test was used for statistical analysis. **p < 0.01.

To evaluate if the implementation of the CBX3 UCOE improves the expression of synthetic cassettes in vivo, we generated transgenic mouse lines in which the P_tet_ promoter controls the expression of luciferase to facilitate imaging in live animals. We integrated the CBX3-flanked P_tet_-luc cassette or the rtTA gene into the Rosa26 locus of ES cells and generated single transgenic mouse lines. By subsequent mating, we established U^R^-Tet-luc/R transgenic mice in which the two homologous Rosa26 loci harbor the CBX3-flanked P_tet_-luc cassette and the rtTA gene, respectively. Corresponding Tet-luc/R mice without CBX3 were generated as control (Figure 3A). Luciferase expression was determined by in vivo bioluminescence imaging (BLI) of mice, which were treated with doxycycline for 21 days or left untreated. Irrespective of the feeding with doxycycline, the luminescence of Tet-luc/R mice was close to the background, indicating silencing of the synthetic cassette in vivo (Figure 3B,C). In contrast to Tet-luc/R mice, U^R^-Tet-luc/R mice showed significantly higher levels of bioluminescence, which increased to 500–1000-fold when the mice had received doxycycline. Expression analysis of organ lysates revealed a limited (threefold) response to doxycycline in the kidney and spleen of U^R^-Tet-luc/R mice, but no increase in the liver (Figure 3D). The lysates of the respective organs from Tet-luc/R mice showed luciferase below the threshold. Thus, while CBX3 facilitated activation of the P_tet_ promoter in U^R^-Tet-Luc/R mice, inducibility upon feeding animals with doxycycline was limited.

### Doxycycline-Controlled Expression in U^R^-Tet-Luc Mice Is Supported by Targeted Demethylation

**Figure 4:**
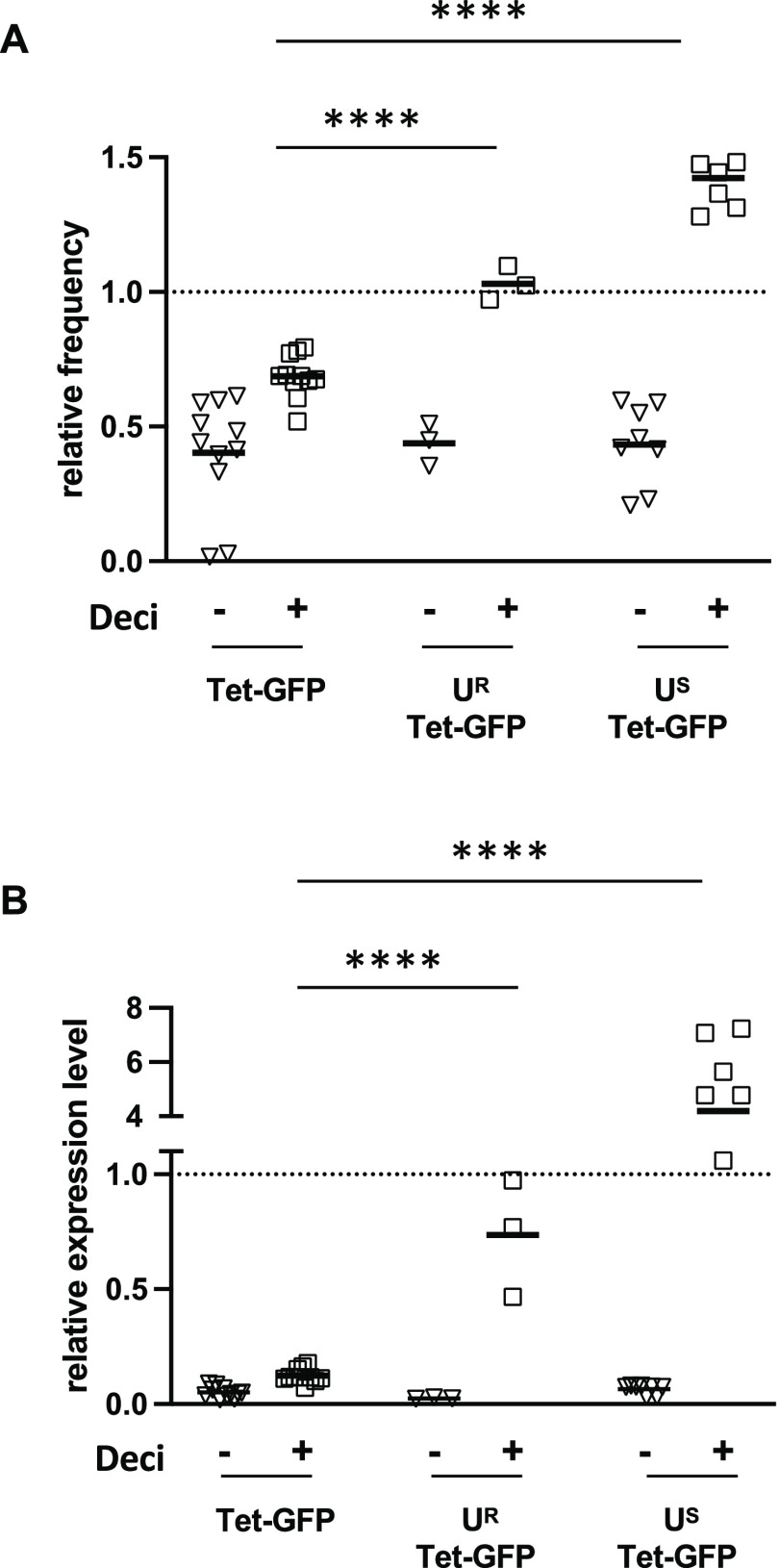
Blocking of DNA methylation restores expression in differentiated U^R^-Tet-GFP and U^S^-Tet-GFP cells. U^R^-Tet-GFP and U^S^-Tet-GFP ES cells were cultivated in the presence of doxycycline (2 μg/mL) and differentiated by cultivation in the absence of LIF for 6 days. Decitabine was added for 3 days prior to flow cytometry analysis. Relative frequency of GFP expressing cells (A) and relative expression levels (MFI) (B) are depicted, in which the respective ES cell population was set to 1 (indicated by the dashed line). Student’s *t*-test was used for comparisons between Tet-GFP and U^S^-Tet-GFP or U^R^-Tet-GFP, respectively, as indicated. ****p < 0.0001. The figure represents data from 2–3 experiments.

We previously demonstrated that silencing of the P_tet_ promoter in the Rosa26 locus is a consequence of excessive methylation of the CpG motifs during differentiation.^13^ Thus, we asked if DNA methylation reduces the activity of the P_tet_ promoter in the context of the CBX3 UCOE. First, we confirmed that the loss of gene expression in differentiated CBX3 targeted cells was associated to DNA methylation (Supporting Information Figure S3). Next, we differentiated U^R^-Tet-GFP, U^S^-Tet-GFP, and control Tet-GFP cells in the presence of decitabine, an inhibitor of DNA methylation. In all the selected samples, GFP expression was completely blocked upon differentiation in the absence of Decitabine. Notably, decitabine increased GFP expression in U^R^-Tet-GFP and U^S^-Tet-GFP cells, while Tet-GFP control cells remained largely unaffected. This suggests that CBX3 supports expression when combined with demethylation (Figure 4A,B).

**Figure 5:**
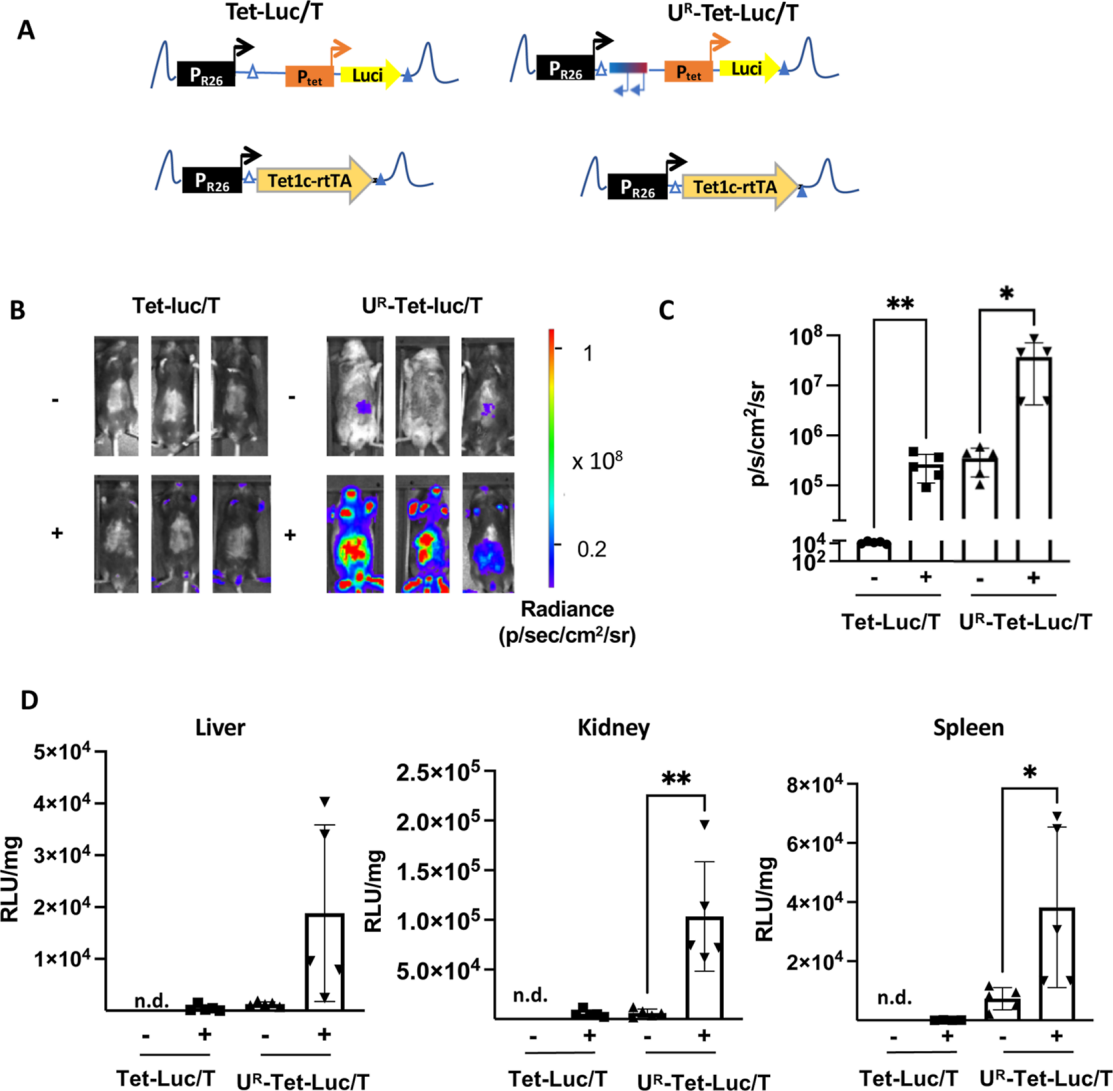
The CBX3 UCOE renders the P_tet_ promoter susceptible to DNA demethylation in vivo. (A) Schematic drawing of the synthetic cassettes targeted into the Rosa26 locus in transgenic mice. Tet-luc/T and U^R^-Tet-Luc/T mice encode a P_tet_ promoter-driven fusion protein of luciferase and GFP integrated into one of the Rosa26 loci as well as a Rosa26 promoter-driven TET1c-rtTA gene in the homologous locus. In U^R^-Tet-Luc/T mice, a reversely orientated CBX3 UCOE is integrated upstream of the P_tet_ promoter. White and blue triangles indicate the heterologous FRT sites used for targeted integration of the cassettes into the murine Rosa26 locus on chromosome 6. (B) Representative in vivo non-invasive BLIs of doxycycline-induced (+) or control (−) Tet-luc/T or U^R^-Tet-luc/T animals. Doxycycline (2 mg/mL) was administered for 3 weeks in drinking water. The color bar indicates photons/cm^2^/s/steradian. (C) Quantification of the BLI of control (−) and luc doxycycline-induced (+) Tet-luc/T and U^R^-Tet-luc/T mice, with five mice per group. Student’s *t*-test was used *p < 0.05; **p < 0.01. (D) Quantification of luciferase expression from organ lysates (liver, kidney, and spleen) of control and doxycycline-induced Tet-luc/T and U^R^-Tet-luc/T mice. RLU were normalized to milligrams of total protein in the tissue sample. The depicted data were derived from 4 Tet-luc/T and 5 U^R^-Tet-luc/T control mice (off) and from 5 Tet-luc/T and 5 U^R^-Tet-luc/T doxycycline mice. Student’s *t*-test was used. *p < 0.05; **p < 0.01. n.d.: all samples were below the detection limit or <1000 RLU/mg.

Previously, we demonstrated that in mice, silencing of the P_tet_ promoter can be reverted by the expression of TET1c-rtTA, a fusion protein of a catalytic domain of the ten-eleven translocation (TET1) demethylase and rtTA, which both demethylates and transactivates the P_tet_ promoter.^13^ Hydrodynamic injection of a TET1c-rtTA-expressing vector was shown to demethylate and reactivate the P_tet_ promoter.^13^ To evaluate if the expression of TET1c-rtTA can improve P_tet_ expression, we targeted the TET1c-rtTA gene into the Rosa26 locus of ES cells and generated a transgenic mouse line. By subsequent mating with the luciferase mouse lines, we generated U^R^-Tet-Luc/T and control Tet-Luc/T mice (Figure 5A). We evaluated the luciferase expression after feeding mice with doxycycline. In U^R^-Tet-Luc/T mice, luciferase expression was overall upregulated and tightly controlled by doxycycline, both in living animals (Figure 5B,C) and in organ lysates (Figure 5D). Notably, the overall BLI levels were about 10-fold higher if compared to U^R^-Tet-Luc/R mice (cf Figure 3). Similarly, also U^R^-Tet-Luc/T showed an elevated luciferase expression in the presence of doxycycline, although expression levels remained low (Figure 5C,D).

Together, the results indicate that both the demethylating factor TET1c-rtTA and the CBX3 UCOE are required for the doxycycline control of the P_tet_ promoter in the Rosa26 locus in transgenic animals. This suggests that the CBX3 UCOE maintains the P_tet_ cassettes in a state that renders the cassettes receptive for demethylation, thereby overcoming silencing.

## Discussion

Few chromosomal integration sites have been explored with regard to their ability to support the expression of doxycycline-dependent expression cassettes in mice. This includes the HPRT locus,^36,37^ the Rosa26 locus,^12−14,38,39^ the collagen locus,^40,41^ as well as few sites which were identified upon random integration and screening.^6^ Depending on the chromosomal site, the cassette design, and the experimental design, varying results were reported ranging from doxycycline-controlled expression^40,41^ to pronounced heterogeneity^6,12,14,38^ or even silencing.^6,13,38,39,42^ This suggests that the chromosomal environment and/or cassette-specific epigenetic constraints can compromise the performance of the synthetic cassettes.

In this study, we investigated if the UCOEs can overcome the expression heterogeneity of P_tet_-driven expression. To this end, we focused on the HNRPA2B1-CBX3 UCOE as well as its previously characterized subfragments A2UCOE and CBX3 and evaluated their potential to stabilize the expression of the synthetic P_tet_ cassettes in murine ES cells, in differentiated cells, as well as in transgenic mice. To avoid unspecific effects arising when comparing the performance of the expression cassettes in different integration sites, we investigated the performance of the expression cassettes within the same chromosomal site, the well-characterized Rosa26 locus on chromosome 6. This integration site is frequently used for the reliable expression of integrated transgenes, with the endogenous Rosa26 promoter driving the transgene expression in virtually all tissues and differentiation states,^9^ and is accessible by classic homologous recombination or advanced targeting methods.^35,43^

We observed that P_tet_-driven expression in the Rosa26 locus was characterized by pronounced cell-to cell variation in isogenic ES cells, resulting in a highly heterogeneous expression phenotype. In contrast to the reported beneficial effect on various constitutive promoters upon integration in hematopoietic cells,^28,29,44^ the larger UCOE fragments HNRPA2B1-CBX3 and A2UCOE reduced the overall expression level and frequency of P_tet_-controlled expression in the Rosa26 locus in ES cells. This observation is in contrast to a previous study in which the A2UCOE did not significantly reduce the expression of the P_tet_ promoter after random integration in mouse pluripotent stem cells.^45^ This suggests that the effect of the 1,5kb spanning A2UCOE on the P_tet_ promoter is negatively modulated in the context of the chromosomal environment of the Rosa26 locus.

A different result was obtained for the small CBX3 fragment cells: while it did not improve the expression properties of P_tet_ cassettes in the Rosa26 locus in ES cells, it could reduce silencing of P_tet_ cassettes, in particular at early states of differentiation. This is in agreement with previous studies demonstrating that the CBX3 element overcomes differentiation-associated silencing of various constitutive promoters in random integration sites^26,28^ as well as in the Rosa26 locus.^27^ Interestingly, in none of these studies could the UCOE completely prevent silencing; rather, a considerable fraction of cells lost expression even in the presence of the UCOE. It remains to be analyzed if this loss of expression is restricted to certain differentiation states or rather occurs stochastically. Notably, although CBX3 comprises endogenous promoter activities,^27,28^ it does not compromise doxycycline-controlled expression of the P_tet_ promoter in the Rosa26 locus in ES cells or after differentiation.

While the CBX3 could reduce silencing of the Rosa26 locus during differentiation, it could not overcome expression heterogeneity between individual cells of the clonal cell population. This suggests fluctuations in the chromatin status of the P_tet_ promoter even in the context of UCOEs. Thus, while the CBX3 UCOE is not sufficient to fully protect from methylation and does not homogenously activate transgene expression in all cells, it is crucial to render the chromatin landscape of the Rosa26 locus susceptible to DNA demethylation.

The implementation of the CBX3 UCOE was also critical to maintain the P_tet_-driven expression in the Rosa26 locus in mice, although the overall levels were low/moderate and doxycycline-mediated control was limited. Similar to the in vitro differentiated cells after the decitabine-mediated block of methylation, in mice, the specific recruitment of the transactivating and demethylating TET1c-rtTA protein to the P_tet_ promoter further improved the expression and inducibility. This demonstrates that although the UCOE can keep the Rosa26 locus open, it cannot fully prevent differentiation-induced silencing of P_tet_ cassettes but rather requires active prevention of DNA methylation.

A recent study observed heterogeneity in P_tet_-controlled expression for so-called “all-in-one” vectors in which the transactivator is expressed from a neighboring constitutive promoter, which could be overcome by increased transactivator expression after targeted integration into the Rosa26 locus.^46^ The results of our studies show that the silencing of P_tet_ cassettes during development also occurs if the two transcription units are separated on different chromosomes and even when the transactivator is expressed from the Rosa26 promoter. This may suggest increased silencing pressure during the remodeling of chromatin during differentiation.

Together, our results indicate that UCOEs are context-dependent, and their effect on chromatin might be affected by the nature of the promoter and/or the nature of the chromosomal site of integration. While the underlying mechanism(s) remain(s) unclear, it is tempting to speculate that UCOEs can take control of various but not all chromatin architectures that are eminent after the integration of expression cassettes. This suggests that—similar to other regulatory elements of expression vectors^47,48^—also UCOEs have to be carefully validated for a specific application, i.e., in combination with the promoter of interest and for a specific chromosomal environment. In light of the recent developments in the field of synthetic biology, our study may contribute to the exploitation of synthetic circuits in animal models or for future therapeutic applications in mammals.

## Methods/Experimental Section

### ES Cell Cultivation, Generation of Rosa26-Targeted ES Cells, and In Vitro Differentiation

Genetically modified ES cells were cultured on irradiated treated murine embryonic fibroblast feeder cells in ESC medium (knock-out DMEM, 15% ES-tested FCS, 1 mM l-glutamine, 0.1 mM non-essential amino acids, 100 U/mL penicillin/streptomycin (all Invitrogen), 100 μM β-mercaptoethanol, and 1 μg/mL LIF. The cells were passaged every 2–3 days using trypsin/EDTA (Invitrogen).

Specific targeting of the Rosa26 chromosomal locus was performed by RMCE in G4B12 cells as previously described.^35^ G4B12 cells^12^ harbor an RMCE-compatible exchange cassette flanked by heterologous Flp recognition target (FRT) sites as well as a silent neomycin phosphotransferase expression unit at the Rosa26 locus. G418-mediated selection was used to isolate targeted isogenic subclones, which were expanded individually. Correct targeting was confirmed by PCR. Tet-GFP and R26-GFP ES cells have been described before.^13.^ For the generation of the UCOE cassettes, the Tet-GFP targeting vector^13^ was linearized with NheI (5′ to the P_tet_ promoter and 3′ to the polyadenylation signal derived from the bovine growth hormone gene). The 4 kb HNRPA2B1-CBX3 UCOE (HindIII-BamHI fragment derived from pMA689, kindly provided by Dr. Michael Antoniou, Kings College London) was integrated after blunting, resulting in H^S/R^-Tet-GFP. For the generation of A^S/R^-Tet-GFP and US/R/D-GFP cells, NheI-flanked A2UCOE and CBX3 subfragments were generated by PCR, using primer pairs (5-TTAATTTGCTAGCTTGTTAGGCCCTCCGCG–5- TTAATTTGCTAGCTTACCGAGGAGACGCCG) and (5-TTAATTTAGCTAGCCCCGGGAGGTGGTCCC–5-TAAATTAAGCTAGCCGGCCCTCCGCGCCTACAGC), respectively.

For the generation of transgenic animals, P_tet_-luc- and CBX3-P_tet_-luc-targeted ES cells were established in which the respective cassettes were flanked with FRT sites by cloning into pEMTAR^35^ and targeted into G4B12 cells as described above.

Differentiation of targeted ES cells was carried out as described previously.^13^ In brief, ES cells were seeded on gelatin-coated wells and cultured for an indicated time without feeders and in the absence of LIF for 4 or 8 days. For decitabine treatment, 1 × 10^4^–1 × 10^5^ ES cells were seeded per well of a gelatin-coated 12-well plate in the presence and absence of doxycycline and first cultured for 3 days in regular media and then for an additional 3 days in media supplemented with the DNA methyltransferase inhibitor decitabine (1 μM, Sigma-Aldrich).

### Luciferase Analysis

In vitro-cultured cells were washed in PBS and harvested in reporter lysis buffer (Promega, E397A). For the evaluation of organ lysates, tissue samples were homogenized using the FastPrep system (MP Biomedicals, Illkirch Cedex, France). Aliquots of lysates were mixed with 100 μL of Beetle Lysis Juice (PJK). Relative light units were detected using the luminometer Lumat LB 9507 (Berthold) and related to the protein content.

### Flow Cytometry

Flow cytometric analysis was performed as described.^13^ In brief, cells were harvested as described above and resuspended in 2% FCS/PBS and analyzed by FACS Symphony A5 (Beckton & Dickinson). The raw data were then analyzed with the software FLOWJo_v10.7.2 (TreeStar).

## Generation of Transgenic Mice

Transgenic mouse lines in which the respective P_tet_ cassettes (see above) or the synthetic transactivator (rtTA or TET-1c-rtTA cloned into pEMTAR^35^) are integrated into the Rosa26 locus were obtained after blastocyst injection of Flp/RMCE-targeted ES cells in the Transgenic Mouse Facility (TGSM) at the Helmholtz Centre for Infection Research. U^R^-Tet-Luc/R and Tet-Luc/R mice (Figure 3) are modified with the P_tet_ cassette (with or without U^R^) on one chromosome 6 allele, while the synthetic transactivator rtTA is controlled by the endogenous Rosa26 promoter on the other allele; U^R^-Tet-Luc/T and Tet-Luc/T mice (Figure 5) carry the P_tet_ cassette (with or without the U^R^) on chromosome 6, and the synthetic transactivator/demethylator TET1c-rtTA is controlled by the endogenous Rosa26 promoter on the homologous chromosome. Mice used in this study were generated from the respective single transgenic animals by breeding.

## Mouse Experiments

All mice used in this study were genotyped to confirm the presence of the respective expression cassette(s) using specific PCR protocols. Doxycycline was administered to mice via supplementing the drinking water at a final concentration of 2 mg/mL for 21 days.

For bioluminescence, Xenogen IVIS 200 was used. Animals were anesthetized in the induction chamber by 2–2.5% isoflurane (Albrecht). Mice were then injected intraperitoneally with 100 μL of luciferin (30 mg/mL in PBS, Synchem OHG) and placed on a heated (37 °C) platform in an acquisition chamber. Anesthesia was maintained by constant administration of isoflurane via nose cones while images were taken. Initially, a gray-scale image was taken in the light-tight chamber. Photons were collected by a sensitive CCD camera, and the signals were overlaid to the gray scale image. Analyses of images was done with the Living Image 4.7.2 (Igor Pro 4.09A) computer program.

## Statistics

The data are displayed as mean and standard deviation using GraphPad Prism 9.0.2, GraphPad Software. The robust CV representing the statistic measure of the distribution around the median was obtained by analysis of flow cytometry data with Flow Jo (v10.6.2).

## Supporting Information Available

Expression analysis of representative ES cell populations targeted with the indicated cassette; ES cells differentiated by cultivation in the absence of LIF for 4 and 8 days; and methylation analysis of CBX3 cassettes upon differentiation (PDF)
