# Supplementary material for: A Ubiquitous Chromatin Opening Element and DNA Demethylation Facilitate Doxycycline-Controlled Expression during Differentiation and in Transgenic Mice
Source: ACS Synth Biol. 2023 Feb 9;12(2):482–91. doi: 10.1021/acssynbio.2c00450 (PMC9942253; doi:10.1021/acssynbio.2c00450)
Supplement: Supplementary file 1 — sb2c00450_si_001.pdf [file sb2c00450_si_001.pdf]

Supplementary information

A ubiquitous chromatin opening element and DNA demethylation facilitate Doxycycline-controlled expression during differentiation and in transgenic mice

Natascha Gödecke[1$], Sabrina Herrmann[1], Viola Weichelt[1#], Dagmar Wirth[1,2*]
[1] RG Model Systems for Infection and Immunity, Helmholtz Centre for Infection Research, 38124 Braunschweig, Germany
[2] Institute of Experimental Hematology, Medical University Hannover (MHH), 30625 Hannover
[$] Current address: Department of Viral Immunology, Helmholtz Centre for Infection Research, 38124 Braunschweig, Germany
[#] Current address: Leibniz Institute DSMZ - German Collection of Microorganisms and Cell Culture, 38124 Braunschweig, Germany
*Corresponding author email: dagmar.wirth@helmholtz-hzi.de

Supplementary Figure S1

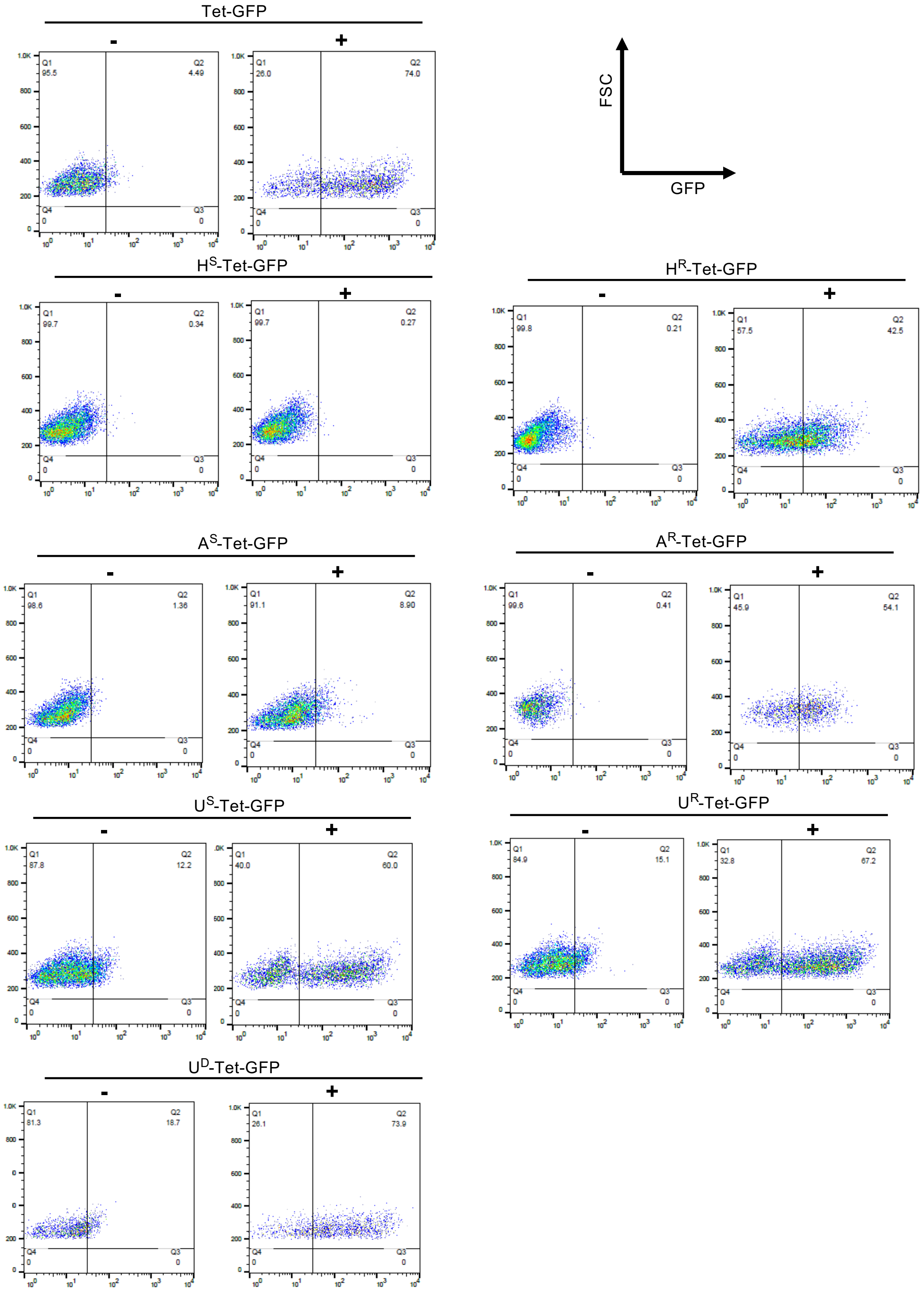

Supplementary Figure S1

Additional information underlying Figure 1. Expression analyses of representative ES cell populations targeted with the indicated cassette. The cell populations were cultivated in absence or presence of 2µg/ml doxycycline for 48h and analysed for GFP expression by flow cytometry.

Supplementary Figure S2

A

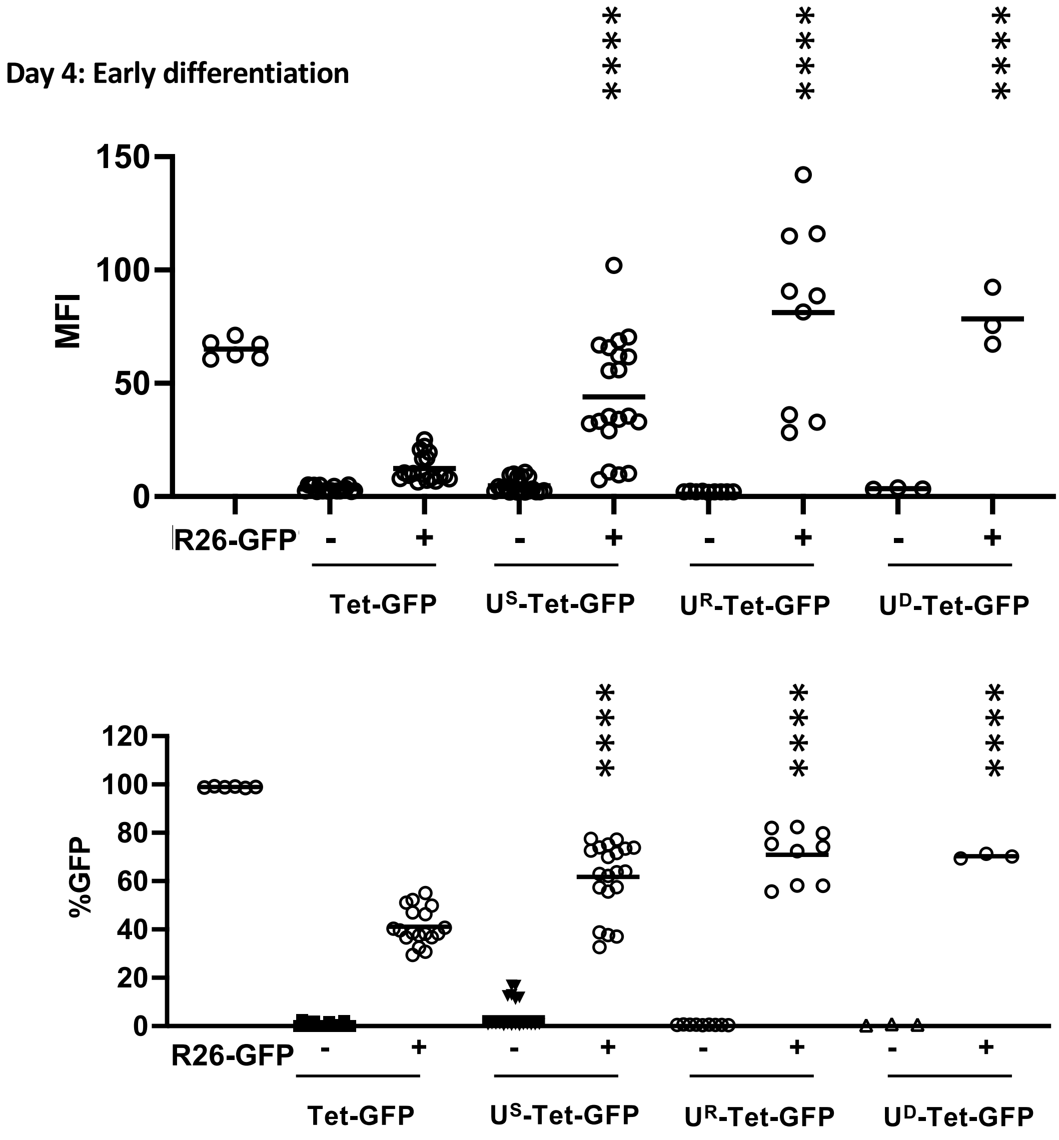

B

Day 8: Late differentiation

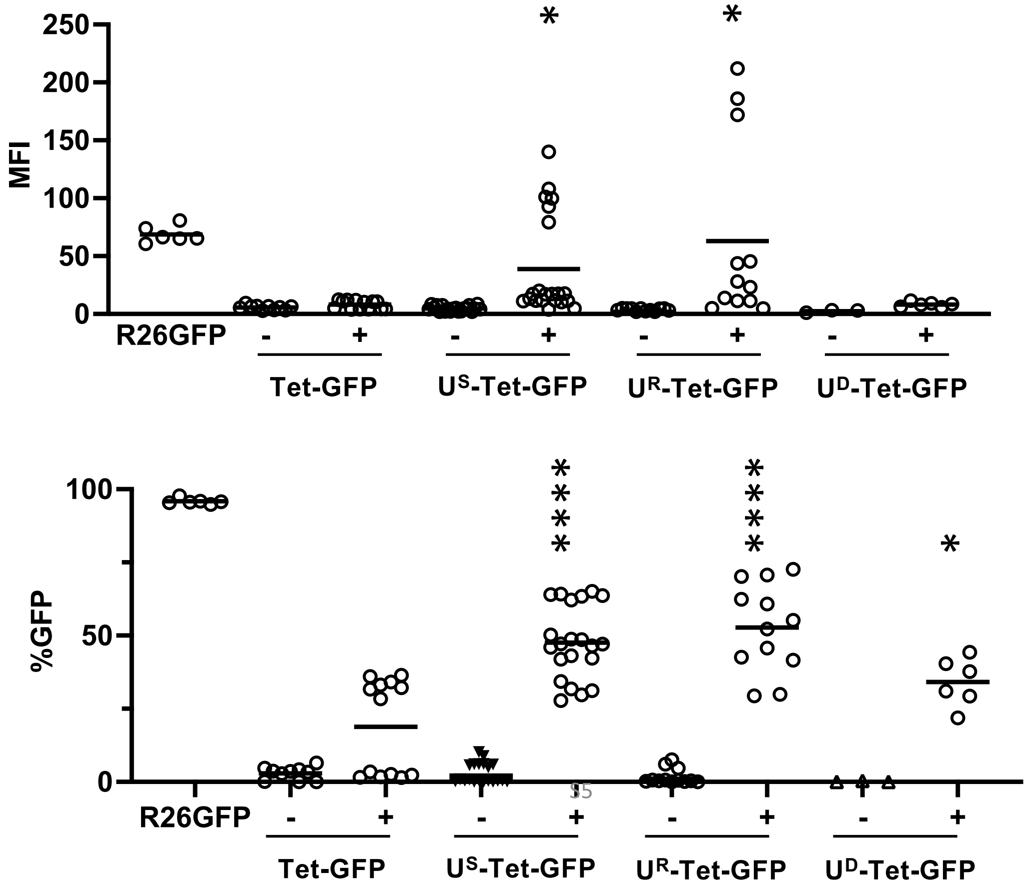

Supplementary Figure S2

Additional information underlying Figure 2. ES cells were differentiated by cultivation in absence of LIF for 4 (A) and 8 days (B). The percentage of GFP expressing cells as well as the median fluorescence intensity (MFI) is plotted. '-' samples were cultured in absence of Doxycycline, '+' samples additionally received doxycycline (2ug/ml) for 48h prior flow cytometry analysis. Statistical analysis: Student's t test was used for comparisons between Tet-GFP (+) and U^S-Tet-GFP, U^R-Tet-GFP or U^D-Tet-GFP, respectively, and is indicated above the respective data set. * $p < 0.05$; **** $p < 0.0001$.

Supplementary Figure S3

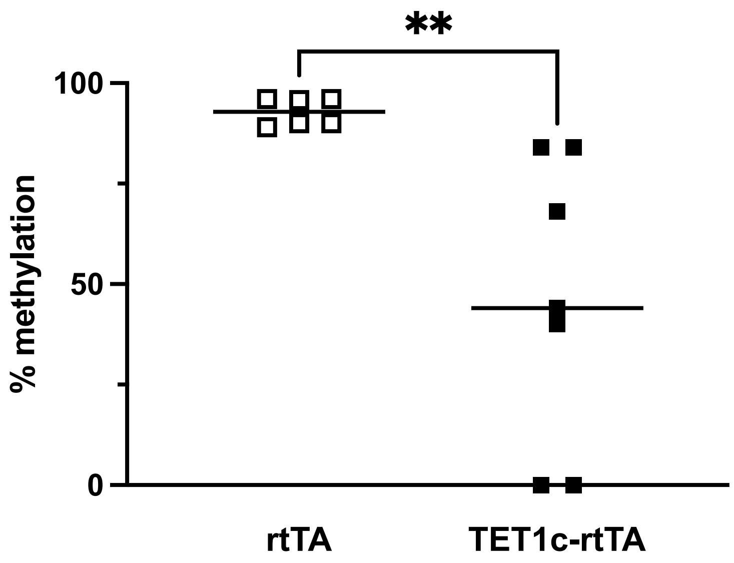

Methylation analysis of cassettes

ES cells targeted with a U^S-Tet-GFP cassette and rtTA or TET1c-rtTA, respectively, were in vitro differentiated. On day 4, genomic from DNA was isolated and used for bisulfite conversion (EpiTect Bisulfit Kit , Qiagen) according to the manufacturer's protocol. The converted DNA was used as template for a semi-nested PCR amplification using forward primer AATTGATTTTTGGTGTATAGGA and reverse primers CTAAAAATTAAAACCCTAAAAAAAA and TGATTTTTGGTGTATAGGAGAG, respectively. PCR fragments were cloned into TOPO TA vector. 7 clones derived from U^S-Tet-GFP cells and 6 clones derived from US-Tet-GFP + TET1c-rtTA were randomly selected. The PCR fragments of these clones was individually sequenced with M13fwd and M13rev primers, .

The frequency of unconverted and converted CpG motifs (representing methylated and non-methylated CpGs, respectively) was determined for each of the fragments/clones. Student's t test was used for statistical analysis. ** $p<0.01$.
